# Correction: The impact of perioperative positive fluid balance on postoperative acute kidney injury in patients undergoing open hepatectomy: A retrospective single center cohort study

**DOI:** 10.1371/journal.pone.0357444

**Published:** 2026-09-01

**Authors:** Natsuda Phothikun, Orapan Pantatong, Maytinee Kulpanun, Somchai Wongpunkamol, Worakitti Lapisatepun, Amarit Phothikun, Warangkana Lapisatepun

All authors should not have been attributed equal contribution to this work.

The affiliation for the seventh author is incorrect. Warangkana Lapisatepun is not affiliated with #4 but with #1: Department of Anesthesiology, Faculty of Medicine, Chiang Mai University, Chiang Mai, Thailand.

## References

[1] Phothikun N, Pantatong O, Kulpanun M, Wongpunkamol S, Lapisatepun W, Phothikun A, et al. The impact of perioperative positive fluid balance on postoperative acute kidney injury in patients undergoing open hepatectomy: A retrospective single center cohort study. PLoS One. 2025;20(4):e0319856. doi: 10.1371/journal.pone.0319856 40168322 PMC11960907

