# Supplementary material for: The impact of perioperative positive fluid balance on postoperative acute kidney injury in patients undergoing open hepatectomy: A retrospective single center cohort study
Source: PLoS One. 2025 Apr 1;20(4):e0319856. doi: 10.1371/journal.pone.0319856 (PMC11960907; doi:10.1371/journal.pone.0319856)
Supplement: S1 Table — (DOCX) [file pone.0319856.s002.docx]

**S1 Table.** Acute kidney injury stage by different balance (Propensity score matched) groups

| Variables | < 1 L Group  (n=193) | 1-2 L Group  (n=193) | > 2 L Group  (n=193) | p |
| --- | --- | --- | --- | --- |
| AKI stage |  |  |  | 0.040 |
| -Stage 1 | 19 (9.84) | 14 (7.25) | 28 (14.51) |  |
| -Stage 2 | 0 (0) | 1 (0.52) | 4 (2.47) |  |
| -Stage 3 | 0 (0) | 0 (0) | 1 (0.52) |  |
|  |  |  |  |  |
| X (X); number (percent %), L; Liters, AKI; Acute kidney injury.  Statistically significant at *p*<0.05 | | | | |

**S2 Table.** Estimated glomerular filtration rate between groups

| Variables | < 1 L Group  (n=193) | 1-2 L Group  (n=193) | > 2 L Group  (n=193) | p |
| --- | --- | --- | --- | --- |
| eGFR |  |  |  |  |
| **Pre-operative** | 80.9 ±27.8 | 82.3±33.8 | 77.0±30.7 | 0.028 |
| **Post-operative** | 75.4±24.9 | 74.6±24.7 | 74.2±26.3 | 0.633 |
|  |  |  |  |  |
| X ± X; Mean ± Standard deviation, L; Liters, eGFR; Estimated glomerular filtration rate (ml/min/1.73m^2^)  Statistically significant at *p*<0.05 | | | | |
