# Supplementary material for: The impact of perioperative positive fluid balance on postoperative acute kidney injury in patients undergoing open hepatectomy: A retrospective single center cohort study
Source: PLoS One. 2025 Apr 1;20(4):e0319856. doi: 10.1371/journal.pone.0319856 (PMC11960907; doi:10.1371/journal.pone.0319856)
Supplement: Attachment — Submitted filename: Response_to_Reviewers_auresp_2.docx [file pone.0319856.s006.docx]

A rebuttal letter.

Dear, Academic Editor

Thank you for giving us the opportunity to submit a 2^nd^ revised draft of our manuscript titled “The Impact of Perioperative Positive Fluid Balance on Postoperative Acute Kidney Injury in Patients Undergoing Open Hepatectomy: A Retrospective Single Center Cohort Study” to PLOS ONE. We appreciate the time and effort you and the reviewers have dedicated to providing valuable feedback on our manuscript. We are grateful to the reviewers for their insightful comments on our paper. We have been able to incorporate changes to reflect most of the suggestions provided by the reviewers.

Here is a point-by-point response to the reviewers’ comments and concerns.

**Comments to the Author**

**Comment from reviewer 2**

**Thank you for your detailed responses to the reviewer's comments on your manuscript titled "The Impact of Perioperative Positive Fluid Balance on Postoperative Acute Kidney Injury in Patients Undergoing Hepatectomy: A Single Center Retrospective Cohort Study."**

**While we appreciate the comprehensive answers provided, we noticed that the corresponding modifications have not been incorporated into the manuscript text. To ensure the reviewer can easily assess the revisions, we kindly request that all changes based on the reviewer's feedback be added directly to the manuscript.**

**Additionally, please highlight these modifications within the manuscript. This will help streamline the review process and ensure clarity regarding the updates made in response to the reviewers' comments.**

**We look forward to receiving the revised manuscript with the highlighted changes at your earliest convenience.**

**Reviewer #2:**

Response: Thank you for pointing this out.

All modifications have been incorporated into the manuscript text. To ensure the reviewer can easily assess the revisions, we have resubmitted the revised manuscript with tracked changes. Additionally, we have provided a version with highlighted modifications for clarity. Revisions based on Reviewer 2’s feedback are marked in green, while those based on Reviewer 3’s feedback are marked in yellow.

**Comment from reviewer 3**

**Thank you once again for inviting me to review this resubmission. The authors are to be commended for their major review. The revised manuscript is much stronger, however there are still substantial minor changes that should be made.**

**Of these, the discussion section requires substantial editing to enhance its clarity, flow, and structure. I have taken the liberty of providing a detailed edit of the discussion, maintaining the writing style of the authors, while improving readability and coherence. Additionally, I have corrected a few factual inaccuracies, particularly concerning the findings of the RELIEF study, to ensure precision and alignment with the evidence.**

**I hope these revisions prove helpful in strengthening the study and making the discussion section more impactful. Overall, this is an impressive and well-executed retrospective study with significant findings that are likely to influence the practice of anesthesia for hepatectomy. I am confident it will be highly cited and contribute meaningfully to the literature.**

**Response**

Thank you for your thoughtful and constructive feedback. We sincerely appreciate your detailed edits to the discussion section, which have greatly improved its clarity, flow, and structure while preserving our writing style. Your corrections, particularly regarding the findings of the RELIEF study, have been invaluable in ensuring the accuracy and alignment of our discussion with the existing evidence.

We have carefully incorporated your suggested revisions and believe they have strengthened the manuscript significantly. Thank you once again for your time and expertise in reviewing our work. Your insightful comments and support are truly appreciated.

**Reviewer #3**

**Comment 3.1: Line 30, Abstract The sentence “….involving 691 patients underwent hepatectomy…” is incomplete. Please change to “A retrospective single-center cohort study was conducted involving 691 patients underwent who underwent an open hepatectomy.”**

Response: Thank you for pointing this out. We revised and updated the manuscript according to your recommendations.

**Comment 3.2: Lines 228-234: Discussion section. This entire paragraph should be deleted. It detracts significantly from the manuscript.**

Response: Thank you for pointing this out. We deleted and updated the manuscript according to your recommendations.

**Comment 3.3: Line 237: Discussion section. The authors state that “For instance, the RELIEF trial compared a liberal fluid regimen to a zero-balance approach during surgery and found a significant increase in AKI rates with the liberal strategy.” This is incorrect! The RELIEF study showed that among patients undergoing major abdominal surgery at increased risk for complications, a restrictive fluid regimen was associated with a higher rate of acute kidney injury.**

Response: Thank you for pointing this out. We revised and updated the manuscript according to your recommendations.

**Comment 3.4: Lines 226-341. Discussion section. The entire discussion section requires substantial editing to enhance its clarity, flow, and structure. I have taken the liberty of providing a detailed edit to maintain your writing style while improving readability and coherence. Additionally, I have corrected a few factual inaccuracies, particularly concerning the findings of the RELIEF study, to ensure precision and alignment with the evidence. I hope these revisions prove helpful in strengthening the study and making the discussion section more impactful. Overall, this is an impressive and well-executed retrospective study with significant findings that are likely to influence the practice of anesthesia for hepatectomy. I am confident it will be highly cited and contribute meaningfully to the literature.**

Response: Thank you for pointing this out. We revised and updated the manuscript according to your recommendations.

**Comment 3.5: Discussion section. Lines 275-279. The authors discuss that fluid overload adversely affects the respiratory system by raising intravascular hydrostatic pressure, leading to pulmonary edema, pleural effusions, and impaired oxygenation. They also discuss damage to the endothelial glycocalyx further heightens susceptibility to infections and sepsis by compromising immune responses (36). Please consider deleting these sentences. This did not investigate pulmonary complications or the immune system in any way. Please update all references accordingly.**

Response: Thank you for pointing this out. We revised and updated the manuscript according to your recommendations.

**Comment 3.6:** Please consider revising the entire discussion to read as follows: (Lines 226-341) …

Response: Thank you for pointing this out. We revised and updated the manuscript according to your recommendations.

We look forward to hearing from you in due time regarding our submission and to responding to any further questions and comments you may have.

Sincerely, yours

Natsuda Phothikun
Department of Anesthesiology, Faculty of Medicine,
Chiang Mai University, Chiang Mai, Thailand
110 Intravaroros Road, Sriphum, Mueng,
Chiang Mai 50200, Thailand

Email: natsuda.dkptk@gmail.com
Tel: +66849090104
