# Supplementary material for: The impact of perioperative positive fluid balance on postoperative acute kidney injury in patients undergoing open hepatectomy: A retrospective single center cohort study
Source: PLoS One. 2025 Apr 1;20(4):e0319856. doi: 10.1371/journal.pone.0319856 (PMC11960907; doi:10.1371/journal.pone.0319856)
Supplement: Attachment — Submitted filename: Response to Reviewers.docx [file pone.0319856.s005.docx]

A rebuttal letter.

Dear, Academic Editor

Thank you for giving us the opportunity to submit a revised draft of our manuscript titled “The Impact of Perioperative Positive Fluid Balance on Postoperative Acute Kidney Injury in Patients Undergoing Open Hepatectomy: A Retrospective Single Center Cohort Study” to PLOS ONE. We appreciate the time and effort you and the reviewers have dedicated to providing valuable feedback on our manuscript. We are grateful to the reviewers for their insightful comments on our paper. We have been able to incorporate changes to reflect most of the suggestions provided by the reviewers.

Here is a point-by-point response to the reviewers’ comments and concerns.

**Comments to the Author**

**Comment from reviewer 1**

**In particular, I found the study very well constructed and with findings that are in line with current literature. Congratulations. The association of acute kidney injury with positive fluid balance needs to be publicized and publicized.**

Response

Thank you for your positive feedback and recognition of our study’s alignment with current literature. We agree that the association between acute kidney injury and positive fluid balance is a crucial topic deserving further attention, and we appreciate the opportunity to contribute to this important discussion.

**Comment from reviewer 2**

**The manuscript titled "The Impact of Perioperative Positive Fluid Balance on Postoperative Acute Kidney Injury in Patients Undergoing Hepatectomy: A Single Center Retrospective Cohort Study" presents an interesting evaluation of the ideal positive fluid balance during hepatectomy to prevent acute kidney injury.**

**After reviewing the manuscript, I have raised several important observations:**

**Reviewer #2:**

**Comment 2.1: How many patients were excluded based on the following criteria: living-donor hepatectomy for liver transplantation, chronic kidney disease stage 5 and end-stage renal disease with renal replacement therapy, and unresolved anemia (hematocrit < 25%) prior to surgery?**

Response:

The exclusion criteria were applied during the initial patient selection. As a result, no patients meeting these criteria were included in the study. While we do not have exact numbers, approximately 30 cases were excluded due to end-stage renal disease or chronic kidney disease stage 5, and around 20 due to living-donor hepatectomy for liver transplantation. The number of cases excluded for unresolved anemia is unknown.

**Comment 2.2: The clinical variables evaluated should be clearly outlined in the Methods section.**

Response: Thank you for pointing this out.

The clinical variables evaluated included patient demographics (gender, age, weight, and BMI), ASA classification, and comorbidities such as diabetes mellitus, hypertension, dyslipidemia, cardiovascular and non-cardiovascular diseases. Hepatic conditions were categorized as benign tumors, hepatocellular carcinoma (HCC), liver metastasis, cholangiocarcinoma (CCA), or other pathologies, alongside hepatitis status (Hepatitis B, Hepatitis C, or co-infection). Liver and kidney function were assessed using the Child-Turcotte-Pugh classification, MELD score, and CKD staging. Intraoperative data included the type of hepatectomy (minor or major), anesthesia and operating times, estimated blood loss, significant intraoperative hypotension, and the use of norepinephrine and colloids. These variables were extracted from medical records and anesthetic charts, with data validated by independent reviewers for accuracy and relevance to perioperative outcomes.

**Comment 2.3: In the Methods section, what variables were used for patient matching in the propensity score matching (PSM)?**

Response: Thank you for pointing this out.

The variables age, gender(male), BMI, CKD staging, and Child-Turcotte-Pugh classification were used for matching.

**Comment 2.4 What criteria were used for selecting variables for the multivariable analysis? Was it based on p<0.05 in the univariable analysis?**

Response: Thank you for pointing this out.

The variables included in the multivariable analysis were primarily selected based on statistical significance in the univariable analysis (p < 0.05). This criterion was used to identify variables with a potential association with the outcome. However, clinical relevance and prior evidence supporting their association with the outcome were also considered to ensure a robust and meaningful multivariable model. For example, variables such as Child-Turcotte-Pugh classification and operation time >5 hours were included due to their strong univariable associations (p < 0.05) and established clinical significance.

This table shows the full multivariable analysis with all variables included.

| Variables | Univariable analysis | | | Multivariable analysis | | |
| --- | --- | --- | --- | --- | --- | --- |
|  | OR | p | 95% CI | OR | p | 95% CI |
| Male | 0.71 | 0.223 | 0.41, 1.23 | 0.77 | 0.372 | 0.44, 1.35 |
| Age >60 year | 0.78 | 0.373 | 0.45, 1.35 | 0.80 | 0.448 | 0.45, 1.41 |
| BMI <18 kg/m^2^ | 0.71 | 0.488 | 0.27, 1.85 | 0.65 | 0.375 | 0.24, 1.70 |
| BMI >25 kg/m^2^ | 0.66 | 0.235 | 0.34, 1.31 | 0.68 | 0.261 | 0.34, 1.34 |
| Albumin < 2.8 g/dl | 1.39 | 0.510 | 0.52. 3.74 | 1.00 | 1.000 | 0.35, 2.84 |
| Child-Turcotte-Pugh |  |  |  |  |  |  |
| A | 0.32 | <0.001 | 0.17, 0.61 | 0.34 | 0.001 | 0.17, 0.65 |
| B | 2.67 | 0.004 | 1.37, 5.16 | 2.55 | 0.007 | 1.29, 5.05 |
| C | 15.7 | 0.025 | 1.41-17.82 | 15.6 | 0.029 | 1.32,18.87 |
| Chronic kidney stage |  |  |  |  |  |  |
| Stage 1 | 1.42 | 0.207 | 0.82, 2.49 | 1.42 | 0.223 | 0.81, 2.53 |
| Stage 2 | 0.98 | 0.949 | 0.59, 1.64 | 1.00 | 0.990 | 0.60, 1.68 |
| Stage 3 | 0.72 | 0.291 | 0.38, 1.33 | 0.70 | 0.273 | 0.37, 1.32 |
| Stage 4 | 0.69 | 0.725 | 0.09, 5.43 | 0.69 | 0.729 | 0.09, 5.55 |
| Operation time > 5 hours | 2.23 | 0.003 | 1.33, 3.91 | 1.79 | 0.044 | 1.01, 3.19 |
| Anesthetic time > 5 hours | 1.88 | 0.045 | 1.01, 3.48 | 1.66 | 0.120 | 0.87, 3.15 |
| Significant Intra-op hypotension | 1.50 | 0.178 | 0.83, 2.71 | 0.95 | 0.912 | 0.45, 2.03 |
| EBL > 500 ml | 1.69 | 0.067 | 0.96, 2.95 | 1.12 | 0.739 | 0.57, 2.18 |
| EBL > 1000 ml | 2.25 | 0.002 | 1.34, 3.77 | 1.86 | 0.026 | 1.08, 3.23 |
| EBL > 2000 ml | 1.69 | 0.229 | 0.72, 3.99 | 0.44 | 0.225 | 0.12, 1.66 |
| Colloid use | 2.13 | 0.028 | 1.08, 4.18 | 1.47 | 0.293 | 0.71, 3.07 |
| Starch usage | 1.45 | 0.182 | 0.84, 2.53 | 1.04 | 0.893 | 0.57, 1.91 |
| Gelatin usage | 1.64 | 0.109 | 0.89, 3.03 | 1.09 | 0.814 | 0.54, 2.17 |
| RBC transfusion | 1.47 | 0.144 | 0.87, 2.46 | 0.86 | 0.643 | 0.45, 1.62 |
| FFP transfusion | 1.21 | 0.512 | 0.68, 2.16 | 0.56 | 0.129 | 0.26, 1.19 |
| Platelet transfusion | 1.18 | 0.829 | 0.26, 5.35 | 0.34 | 0.250 | 0.05, 2.15 |
| Intra-op norepinephrine | 2.45 | 0.006 | 1.29, 4.64 | 1.78 | 0.101 | 0.89, 3.58 |
|  |  |  |  |  |  |  |
| OR; Odd ratio, CI; Confident interval, Kg; Kilogram, m; meter, ml; milliliter, BMI; Body Mass Index, EBL; Estimated blood loss, intra-op; intra operative, RBC; Red blood cell, FFP; Fresh frozen plasma.  Statistically significant at p <0.05. | | | | | | |

**Comment 2.5: In the Discussion section, the authors mention the potential impact of incomplete or invalid data due to the retrospective nature of the study. Could you clarify which specific data were missing and how many patients were lost for each variable?**

Response: Thank you for pointing this out.

While this retrospective study examined a considerable number of patients and adhered to gold standard criteria for AKI assessment, including the use of propensity scores to minimize patient heterogeneity, it also had some limitations. Firstly, due to its retrospective nature, certain key data points were incomplete or missing, particularly those related to the primary outcome. For example, cases with uncertain records of AKI diagnosis or incomplete documentation of postoperative creatinine levels accounted for 13.84% of the dataset (n = 111 patients) (starting with total 802 case). These cases were excluded from the final analysis, which may have introduced selection bias and impacted the reliability of the findings. Secondly, using a database from a single center might limit how applicable the results are to broader populations. Lastly, certain data points, such as estimations of intraoperative bleeding, were reliant on individual practices and could have varied among attending anesthesiologists, potentially affecting the study's outcomes.

A complete case analysis (non-imputation) approach was utilized. Imputation was deemed unnecessary, as thorough data exploration revealed that essential data had a missing rate of less than 10%. Specifically, for diagnosis variables, the missing rate was exceptionally low at 0.17%, while all other variables had no missing data after PS matching.

| Variable | Missing | Total | Percent Missing |  | Variable | Missing | Total | Percent Missing |
| --- | --- | --- | --- | --- | --- | --- | --- | --- |
| Gender | 0 | 579 | 0 |  | Intake | 0 | 579 | 0 |
| Age | 0 | 579 | 0 |  | Output | 0 | 579 | 0 |
| Wtkg | 0 | 579 | 0 |  | balance | 0 | 579 | 0 |
| Htcm | 0 | 579 | 0 |  | hypotension | 0 | 579 | 0 |
| BMI | 0 | 579 | 0 |  | Ephedrine | 0 | 579 | 0 |
| ASA | 0 | 579 | 0 |  | NE | 0 | 579 | 0 |
| Diagnosis | 1 | 579 | 0.17 |  | Epinephrine | 0 | 579 | 0 |
| Hepatitis | 0 | 579 | 0 |  | Dopamine | 0 | 579 | 0 |
| ChildPH | 0 | 579 | 0 |  | Dobutamine | 0 | 579 | 0 |
| +UD | 0 | 579 | 0 |  | Reverse | 0 | 579 | 0 |
| DM | 0 | 579 | 0 |  | Extubation | 0 | 579 | 0 |
| HTN | 0 | 579 | 0 |  | hospstayd | 0 | 579 | 0 |
| DLP | 0 | 579 | 0 |  | Intubation~d | 0 | 579 | 0 |
| UDCardio | 0 | 579 | 0 |  | Postophosp~d | 0 | 579 | 0 |
| preHb | 0 | 579 | 0 |  | Reop | 0 | 579 | 0 |
| preHct | 0 | 579 | 0 |  | Deathinadm | 0 | 579 | 0 |
| preWBC | 0 | 579 | 0 |  | momortality | 0 | 579 | 0 |
| prePlt | 0 | 579 | 0 |  | Stroke | 0 | 579 | 0 |
| preBUN | 0 | 579 | 0 |  | delirium | 0 | 579 | 0 |
| preCr0 | 0 | 579 | 0 |  | Atelect | 0 | 579 | 0 |
| AKI | 0 | 579 | 0 |  | Pneumonia | 0 | 579 | 0 |
| AKIstage | 0 | 579 | 0 |  | Pleuraleff | 0 | 579 | 0 |
| Alb | 0 | 579 | 0 |  | PE | 0 | 579 | 0 |
| ALP | 0 | 579 | 0 |  | Hypotension | 0 | 579 | 0 |
| AST | 0 | 579 | 0 |  | Ileus | 0 | 579 | 0 |
| ALT | 0 | 579 | 0 |  | UTI | 0 | 579 | 0 |
| DB | 0 | 579 | 0 |  | SSI | 0 | 579 | 0 |
| TB | 0 | 579 | 0 |  | DVT | 0 | 579 | 0 |
| Anestimemin | 0 | 579 | 0 |  | ARDS | 0 | 579 | 0 |
| Optimemin | 0 | 579 | 0 |  | Liverfailure | 0 | 579 | 0 |
| crystalloid | 0 | 579 | 0 |  | CardiacArrest | 0 | 579 | 0 |
| Colloid | 0 | 579 | 0 |  | stageCKD | 0 | 579 | 0 |
| Stratch | 0 | 579 | 0 |  | CKD | 0 | 579 | 0 |
| Gelatin | 0 | 579 | 0 |  | HD | 0 | 579 | 0 |
| Albumin | 0 | 579 | 0 |  | POsepsis | 0 | 579 | 0 |
| PRCtrans | 0 | 579 | 0 |  | Pobleed | 0 | 579 | 0 |
| FFPtrans | 0 | 579 | 0 |  | posbalance | 0 | 579 | 0 |
| plttrans | 0 | 579 | 0 |  | eGFR0 | 0 | 579 | 0 |
| EBL | 0 | 579 | 0 |  | eGFR1 | 0 | 579 | 0 |
| UOPml | 0 | 579 | 0 |  |  |  |  |  |

**Comment from reviewer 3**

**Thank you for the opportunity to review the manuscript by Phothikun et al., titled “The Impact of Perioperative Positive Fluid Balance on Postoperative Acute Kidney Injury in Patients Undergoing Hepatectomy: A Single-Center Retrospective Cohort Study.” I commend the authors for addressing a clinically significant issue in perioperative management of hepatectomy patients. This study is well-conceived, leveraging a large sample size to explore the relationship between fluid balance and postoperative acute kidney injury (AKI).**

**While the study provides valuable insights, there are several methodological and reporting issues that require attention to enhance the validity and clarity of the findings. I hope the following comments and suggestions will help refine the manuscript and improve its competitiveness for publication in PLOS ONE.**

**Reviewer #3**

**Comment 3.1: The Background section could be restructured for greater impact. Consider rewording as follows:
“Low central venous pressure (CVP) or fluid restriction strategies are frequently employed during liver parenchymal resection to minimize intraoperative blood loss. However, both hypovolemia and excessive fluid administration can impair organ perfusion, increasing the risk of renal dysfunction and acute kidney injury (AKI). This study explores the relationship between perioperative fluid management strategies and renal outcomes in patients undergoing hepatectomy.”**

Response: Thank you for pointing this out. We revised and updated the manuscript according to your recommendations.

**Comment 3.2: Add the term “single-center” between “retrospective” and “cohort.”**

Response: Thank you for pointing this out. We revised and updated the manuscript according to your recommendations.

**Comment 3.3: Insert a period at the end of the last sentence in the Methods section.**

Response: Thank you for pointing this out. We revised and updated the manuscript according to your recommendations.

**Comment 3.4: Line 79: Replace “There were many different strategies for fluid therapy during hepatectomy” with “Numerous strategies for fluid intervention during hepatectomy have been reported.”**

Response: Thank you for pointing this out. We revised and updated the manuscript according to your recommendations.

**Comment 3.5: Line 80: This sentence is incomplete and needs correction.**

Response: Thank you for pointing this out. We revised and updated the manuscript according to your recommendations.

**Comment 3.6: Lines 81-82: Ensure consistent use of abbreviations after their first introduction. Replace “central venous pressure” with CVP throughout.**

Response: Thank you for pointing this out. We revised and updated the manuscript according to your recommendations.

**Comment 3.7: Line 88: Revise to “However, patients with a positive fluid balance experienced more complications and higher mortality rates [19].”**

Response: Thank you for pointing this out. We revised and updated the manuscript according to your recommendations.

**Comment 3.8: Lines 90-93: Consider the following rewording for clarity: “The optimal fluid therapy strategy during hepatectomy remains unclear, and the impact of varying fluid volumes on kidney function and the incidence of postoperative AKI has not been well-defined. This study aims to investigate the association between perioperative positive fluid balance and postoperative AKI in hepatectomy patients.”**

Response: Thank you for pointing this out. We revised and updated the manuscript according to your recommendations.

**Comment 3.9: Clarify why patients with ASA class IV were included in Table 1 despite the stated exclusion criteria (ASA I-III). Five such patients are noted in the database. Revise or comment.**

Response: Thank you for pointing this out.

We initially planned to include only patients with ASA classes I to III in the study. However, after analyzing the results, we found no significant differences between the study groups when ASA class IV patients were included. Therefore, these patients were ultimately included in the study, although the inclusion criteria were not updated to reflect this adjustment. We revised and updated the manuscript according to your recommendations.

**Comment 3.10: Address the inclusion of patients with Child-Turcotte-Pugh (CTP) class C cirrhosis, despite the general contraindication for elective major liver resection in these cases. Provide context or justification using evidence-based references, such as Suman & Carey (2006). The authors conclude that patients with Child-Turcotte Pugh class C undergoing major liver resection and Child-Turcotte-Pugh scores were significantly associated with an increased risk of post-hepatectomy AKI. Patients with Child-Turcotte-Pugh (CTP) class C cirrhosis face significantly elevated perioperative mortality rates when undergoing major liver resection. Most centres would not undertake major liver resection surgery with patients with CTP class C. Studies indicate that these patients experience mortality rates exceeding 50% in the short term. For instance, a systematic review reported a short-term mortality rate of 52% (95% confidence interval: 33.5–70.0%) for CTP class C patients undergoing cardiac surgery. Similarly, another study found that patients with CTP class C cirrhosis had a postoperative mortality rate above 70% following abdominal surgery. Given these substantial risks, elective major liver resection is generally contraindicated in patients with Child-Turcotte-Pugh class C cirrhosis. (See Suman A, Carey WD. Assessing the risk of surgery in patients with liver disease. Cleve Clin J Med. 2006 Apr;73(4):398-404. doi: 10.3949/ccjm.73.4.398. PMID: 16610401.) Please comment?**

Response: Thank you to the reviewer for raising this important concern, which may indeed cause confusion among readers.

In principle, the Child-Turcotte-Pugh (CTP) score was originally designed to evaluate chronic liver disease in patients without complicating factors such as obstructive jaundice. In our cohort, however, several patients particularly those with cholangiocarcinoma (CCA) or hepatocellular carcinoma (HCC) with bile duct tumor thrombus presented with preoperative obstructive jaundice. In these cases, where major hepatectomy with hilar resection or tumor thrombectomy was mostly required, elevated total bilirubin and decreased albumin levels may have artificially inflated the CTP score, inaccurately classifying certain patients as CTP class C. Therefore, their high CTP scores did not necessarily reflect the full extent of intrinsic liver dysfunction, but rather the impact of biliary obstruction.

Furthermore, the major hepatectomy typically requires total bilirubin levels to be below 3 mg/dL before proceeding. However, some patients with total bilirubin exceeding 3 mg/dL, even after preoperative drainage, still underwent resection due to the urgent for cancer treatment. We acknowledge that major liver resection in patients with a true CTP class C cirrhosis is associated with high perioperative risk and is generally contraindicated, as outlined by Suman and Carey (2006). Nonetheless, in those with obstructive jaundice, the decision to operate must also take into account the potential for improving hepatic function after biliary decompression, as well as the oncological necessity of resecting liver tumor. Our findings underscore the importance of careful preoperative assessment, judicious patient selection, and meticulous perioperative management in such complex scenarios.

**Comment 3.11: The database shows discrepancies: 11 patients with CTP class C cirrhosis are categorized as ASA class II. This appears inconsistent. Please comment on data accuracy and integrity.**

Response: Thank you for pointing this out.

We rechecked the data to ensure accuracy and integrity and found that patients with Child-Turcotte-Pugh (CTP) Class C were incorrectly categorized. Based on the ASA classification criteria, ASA Class IV represents "a patient with severe systemic disease that is a constant threat to life." Therefore, all CTP Class C patients should be categorized under ASA Class IV.

Following a thorough reevaluation, we corrected the data, accordingly, resulting in the updated distribution:

|  | CTP A | CTP B | CTP C | Total |
| --- | --- | --- | --- | --- |
| ASA class 1 | 99 | 2 | 0 | 101 |
| ASA class 2 | 427 | 68 | 0 | 495 |
| ASA class 3 | 62 | 13 | 0 | 75 |
| ASA class 4 | 5 | 0 | 15 | 20 |
| Total | 593 | 83 | 15 | 691 |

|  | All data | | | | Propensity score matching | | | |
| --- | --- | --- | --- | --- | --- | --- | --- | --- |
| Variables | < 1 L Group  (n=213) | 1-2 L Group  (n=261) | > 2 L Group  (n=217) | p | < 1 L Group  (n=193) | 1-2 L Group  (n=193) | > 2 L Group  (n=193) | p |
| ASA Class |  |  |  | 0.816 |  |  |  | 0.794 |
| I | 31 (14.6) | 38 (14.6) | 32 (14.7) |  | 24 (12.4) | 24 (12.4) | 32 (16.6) |  |
| II | 154 (72.3) | 187 (71.6) | 154 (71.0) |  | 143 (74.1) | 147 (76.2) | 137 (71.0) |  |
| III | 25 (11.7) | 26 (10.0) | 24 (11.1) |  | 21 (10.9) | 24 (12.4) | 21 (10.9) |  |
| IV | 4 (1.4) | 10 (3.8) | 7 (3.2) |  | 1 (0.5) | 2 (1.0) | 3 (1.5) |  |

These corrections ensure the accuracy and reliability of the dataset for analysis.

**Comment 3.12: Define the criteria used to classify resections as major or minor**

Response: Thank you for pointing this out.

The major liver resection was defined as resection of four or more liver segments vice versa.

*Reference: Reddy SK, A standard definition of major hepatectomy: resection of four or more liver segments. HPB (Oxford). 2011 Jul;13(7):494-502. doi: 10.1111/j.1477-2574.2011.00330. x. PMID: 21689233; PMCID: PMC3133716.*

**Comment 3.13: Provide a clear definition for “significant intraoperative hypotension.”**

Response: Thank you for pointing this out.

There is increasing evidence to suggest that even transient episodes of systolic blood pressure (SBP) below 100 mmHg and mean arterial pressure (MAP) below 60–70 mmHg may have detrimental effects during non-cardiac surgery.

Intraoperative hypotension (IOH) has been defined variably across the literature, with most studies relying on absolute thresholds or relative deviations from baseline blood pressure values. Nevertheless, there is considerable heterogeneity in how IOH is reported, with definitions often varying between surgical specialties. In this study, IOH was defined using absolute criteria, specifically as MAP < 60–70 mmHg or SBP < 100 mmHg.

*Ref: Weinberg et al. BMC Anesthesiology (2022) 22:69*

**Comment 3.14: Explicitly outline the method for calculating “estimated blood loss.”**

Response: Thank you for pointing this out.

In our study, anesthesiologists primarily use visual estimation for blood loss quantification by assessing blood in suction buckets, gauze, and visible intraoperative loss. Although simple and rapid, this method is subjective and prone to bias. To improve accuracy, we occasionally supplement it with the gravimetric method, which involves weighing sponges and gauze before and after use to calculate absorbed blood, and the hemoglobin balance method, which assesses changes in the patient’s hemoglobin levels pre- and post-operatively. These techniques are used simultaneously to provide more reliable measurements.

*Reference: Ján Zajak, Methods of Blood Loss Quantification in Major Abdominal Surgery: A Narrative Review*

**Comment 3.15: Define the term “Non-CVS” as listed in Table 1.**

Response: Thank you for pointing this out.

In Table 1, "Non-CVS" refers to underlying diseases that do not involve the cardiovascular system. Our review of the database indicates that "non-CVS" primarily includes respiratory conditions, such as chronic obstructive pulmonary disease (COPD) and asthma, as well as a few endocrine disorders, such as thyroid disease.

**Comment 3.16: Discuss why the Charlson Comorbidity Index (CCI) was not used for risk stratification. As a more objective measure than the ASA score, the CCI may influence outcomes. Could its omission bias findings?**

Response: Thank you for pointing this out.

Although the Charlson Comorbidity Index (CCI) is one of the most widely utilized and objective scoring systems for assessing comorbidities that may influence surgical outcomes, the American Society of Anesthesiologists (ASA) has developed the ASA Physical Status Classification System to evaluate a patient’s general health status prior to surgery and anesthesia. This classification system is extensively employed by anesthesiologists to assess a patient’s fitness for surgery and guide anesthetic management throughout the procedure. It is crucial to note, however, that the ASA classification does not predict surgical or anesthetic risks per se; rather, it provides a standardized framework for anesthetists to communicate the patient’s overall health status to other healthcare providers involved in the perioperative care.

In our institution, anesthesiologists play a major role in preoperative evaluation, and as such, the ASA classification remains a key component of this assessment. Consequently, it is consistently documented in patient records, often with greater emphasis than the CCI.

*Reference: Guo R. Correlation of ASA Grade and the Charlson Comorbidity Index with Complications in Patients After Transurethral Resection of Prostate. Urology. 2016 Dec; 98:120-125. doi: 10.1016/j.urology.2016.07.025.*

*G. KILINÇ, the role of ASA score and Charlson comorbidity index in predicting in-hospital mortality in geriatric hip fracture patients, 2023; 27: 7065-7072.*

**Comment 3.17: Provide baseline data for creatinine, hemoglobin, albumin, and bilirubin values.**

Response: Thank you for pointing this out. We updated the manuscript according to your recommendations.

|  | All data | | | | Propensity score matching | | | |
| --- | --- | --- | --- | --- | --- | --- | --- | --- |
| Variables | < 1 L Group  (n=213) | 1-2 L Group  (n=261) | > 2 L Group  (n=217) | p | < 1 L Group  (n=193) | 1-2 L Group  (n=193) | > 2 L Group  (n=193) | p |
| Creatinine | 0.90 ±0.38 | 0.89 ± 0.26 | 0.91 ±0.29 | 0.611 | 0.91 ±0.4 | 0.91 ±0.3 | 0.92 ±0.3 | 0.886 |
| Hemoglobin | 12.65 ±1.8 | 12.32 ±1.8 | 12.23 ±2.2 | 0.063 | 12.67 ±1.8 | 12.47 ±1.9 | 12.27 ±2.3 | 0.153 |
| Albumin | 4.03 ±2.55 | 3.80 ±0.61 | 3.68 ±0.65 | 0.044 | 4.04 ±2.67 | 3.81 ±0.61 | 3.73 ±0.63 | <0.001 |
| Bilirubin | 1.48 ±5.10 | 1.45 ±2.87 | 1.95 ±2.88 | 0.272 | 1.52 ±5.23 | 1.20 ±2.23 | 1.38 ±1.65 | <0.001 |

**Comment 3.18: Report the proportion of patients in each group receiving red blood cell transfusions, fresh frozen plasma (FFP), cryoprecipitate, or platelets.**

Response: Thank you for pointing this out. We updated the manuscript according to your recommendations.

We have thoroughly reviewed the dataset and confirmed that no cryoprecipitate data were available or had been corrected.

|  | All data | | | | Propensity score matching | | | |
| --- | --- | --- | --- | --- | --- | --- | --- | --- |
| Variables | < 1 L Group  (n=213) | 1-2 L Group  (n=261) | > 2 L Group  (n=217) | p | < 1 L Group  (n=193) | 1-2 L Group  (n=193) | > 2 L Group  (n=193) | p |
| RBC transfusion | 37 (17.4) | 73 (28.0) | 129 (59.5) | <0.001 | 34 (17.6) | 52 (26.9) | 118 (61.1) | <0.001 |
| FFP transfusion | 24 (11.3) | 39 (14.9) | 103 (47.5) | <0.001 | 20 (10.4) | 30 (15.5) | 87 (45.1) | <0.001 |
| Platelets transfusion | 5 (2.4) | 4 (1.5) | 9 (4.2) | 0.218 | 4 (2.1) | 2 (1.0) | 9 (4.7) | 0.098 |

**Comment 3.19: Include length of hospital stay, mortality rates, and 30-day readmission rates for each group.**

Response: Thank you for pointing this out. We updated the manuscript according to your recommendations.

We have thoroughly reviewed the dataset and confirmed that no 30-day readmission data were available or had been corrected.

|  | All data | | | | Propensity score matching | | | |
| --- | --- | --- | --- | --- | --- | --- | --- | --- |
| Variables | < 1 L Group  (n=213) | 1-2 L Group  (n=261) | > 2 L Group  (n=217) | p | < 1 L Group  (n=193) | 1-2 L Group  (n=193) | > 2 L Group  (n=193) | p |
| Hospital stays (days) | 10.4 ±8.5 | 10.7 ±9.4 | 11.3 ±9.5 | 0.228 | 10.3 ±8.6 | 10.5 ±7.4 | 11.1 ±9.6 | 0.001 |
| Mortality 30 days | 4 (1.88) | 7 (2.68) | 4 (1.84) | 0.847 | 4 (2.07) | 3 (1.55) | 4 (2.07) | 1.000 |
| Overall Mortality | 7 (3.29) | 10 (3.83) | 6 (2.76) | 0.803 | 7 (3.63) | 5 (2.59) | 6 (3.11) | 0.954 |

**Comment 3.20: Discuss whether fluid balance correlated with adverse events, such as bile leaks, hepatic insufficiency, respiratory complications, bleeding, return to the operating room, sepsis, or wound infections.**

Response:

Excessive fluid administration can lead to hypervolemia, resulting in increased portal venous pressure and exacerbation of hepatic congestion. This heightened pressure causes sinusoidal congestion and dilation of sinusoidal fenestrae, promoting the exudation of protein and fluid into the space of Disse. The accumulation of this exudate impairs the diffusion of oxygen and nutrients to hepatocytes, creating an ischemic environment that compromises liver function and the integrity of biliary anastomoses, thereby increasing the risk of bile leaks and hepatic insufficiency. Similarly, inadequate fluid balance may result in liver hypoperfusion, further impairing hepatic function and overall recovery.

Fluid overload also has significant effects on the respiratory system, primarily due to elevated intravascular hydrostatic pressure. This pressure stimulates the release of atrial natriuretic peptides, leading to damage of the pulmonary endothelial glycocalyx—a vital barrier that preserves vascular integrity. Disruption of the glycocalyx results in fluid leakage into the pulmonary interstitium, causing pulmonary edema, pleural effusions, and impaired oxygenation. Furthermore, the damaged glycocalyx increases susceptibility to infection and sepsis by compromising its protective functions, facilitating microbial infiltration, and weakening immune responses. These effects are particularly concerning in patients with pre-existing organ dysfunctions, as both hepatic and respiratory complications compound their risks of adverse outcomes.

*Reference: Giallourakis CC, The liver in heart failure. Clin Liver Dis 2002; 6:947‐967, viii‐ix., Safran AP. Chronic passive congestion of the liver in man. Electron microscopic study of cell atrophy and intralobular fibrosis. Am J Pathol 1967; 50:447‐463., Ref. https://doi.org/10.1016/bs.ctm.2018.08.005*

**Comment 3.21: Discuss what the unit's fluid management stategy is for patients undergoing hepatectomy. Is "low CVP" used as standard? Do patients receive advanced hemodynamic monitoring? If so, what protocol is applied?**

Response: Thank you for pointing this out.

Current evidence from Anesthesiology Reports 2024 emphasizes the importance of goal-directed fluid management during liver surgery. This approach consists of two distinct phases of fluid management: the fluid restrictive phase during parenchymal transection and the resuscitative phase after resection. During the fluid restrictive phase, relative hypovolemia is deliberately tolerated to reduce hemorrhage and improve surgical exposure. After the resection is complete, the anesthesiologist should return to more typical hemodynamic goals, involving a more liberal fluid resuscitation strategy.

During the fluid restrictive phase, hemodynamic targets include a mean arterial pressure (MAP) of at least 65 mmHg, pulse pressure variation (PPV) or stroke volume variation (SVV) between 10 and 20%, and a central venous pressure (CVP) of less than 5 mmHg, if CVP can be monitored. If hypotension occurs during this phase, the use of vasopressors is recommended as the first line of treatment, as fluid restriction often necessitates moderate doses of these agents. Fluid administration should be minimized as much as tolerated, with the replacement of surgical blood loss being sufficient to maintain hemodynamic stability. Albumin or blood products may be considered if needed to support hemodynamics during this phase.

In the resuscitative phase, or after resection, the anesthesiologist's goal is to restore typical hemodynamic parameters. The recommended MAP is at least 65 mmHg, and PPV or SVV should be reduced to less than or equal to 12%. CVP, however, should not be used to guide resuscitation during this phase. Fluid boluses become the first priority, with liberal use of balanced crystalloids or albumin. For minor resections, fluid administration of 1–2 liters is typically sufficient, whereas major resections may require 2–3 liters or more. In this phase, concurrent correction of coagulopathy and anemia is also emphasized.

The low CVP technique has been a mainstay of anesthesia management in liver resection for at least 25 years. While the approach aims to reduce blood loss, data on its ability to improve postoperative morbidity and mortality remain conflicting. Low CVP targets can be achieved through multiple methods, including restrictive intravenous fluid administration, hypoventilation to reduce intrathoracic pressure, adjustments to body positioning such as Trendelenburg or reverse Trendelenburg, and the use of vasodilators like nitroglycerin or milrinone. Anesthetic techniques, including the use of volatile agents, opioids, or epidural infusions, can also contribute. The combination of these techniques can be individualized, although the optimal approach remains uncertain. It is important to balance the advantages of low CVP against the potential risks of systemic hypotension.

CVP monitoring remains one option for guiding fluid management, but it is not the only standard. Arterial waveform analysis and stroke volume–guided approaches, as part of goal-directed therapy, have emerged as promising alternatives. Arterial waveform analysis with continuous monitoring of SVV or PPV during liver resection appears to be a safe and effective substitute for the traditional low CVP-guided strategy. This approach may reduce the need for routine central venous catheterization, potentially lowering complications and costs. An arterial line is often warranted during major liver resections, as it carries minimal risk and cost relative to the benefits of enhanced monitoring.

This retrospective data indicates that CVP was predominantly used for monitoring in liver resections over the past decade. In recent years, advanced tools that evaluate SVV and cardiac output (CO) via heart-lung interactions from central line and arterial waveform monitoring have been increasingly adopted. However, the documentation of advanced hemodynamic monitoring remains incomplete in many anesthetic records, making it difficult to fully evaluate its impact on outcomes.

*Reference: Marcus, S.G., Syed, S., Anderson, A.L. et al. Goal-Directed Therapy in Liver Surgery. Curr Anesthesiol Rep 14, 197–208 (2024). https://doi.org/10.1007/s40140-024-00613-4*

**Comment 3.22: Confirm whether there were any missing data in this retrospective study. If so:**

**o Identify the type of missingness (MCAR, MAR, MNAR).**

**o Explain how missing data were handled.**

**o Detail propensity-matching strategies, such as imputation or weighting.**

Response: Thank you for pointing this out.

Type of missingness: MACR

The missing data occurred randomly and is not dependent on any factors. The missingness of the "Diagnosis" data does not depend on the missing values themselves and may have resulted from accidental oversight during data collection.

A complete case analysis (non-imputation) approach was utilized. Imputation was deemed unnecessary, as thorough data exploration revealed that essential data had a missing rate of less than 10%. Specifically, for diagnosis variables, the missing rate was exceptionally low at 0.17%, while all other variables had no missing data after PS matching.

| Variable | Missing | Total | Percent Missing |  | Variable | Missing | Total | Percent Missing |
| --- | --- | --- | --- | --- | --- | --- | --- | --- |
| Gender | 0 | 579 | 0 |  | Intake | 0 | 579 | 0 |
| Age | 0 | 579 | 0 |  | Output | 0 | 579 | 0 |
| Wtkg | 0 | 579 | 0 |  | balance | 0 | 579 | 0 |
| Htcm | 0 | 579 | 0 |  | hypotension | 0 | 579 | 0 |
| BMI | 0 | 579 | 0 |  | Ephedrine | 0 | 579 | 0 |
| ASA | 0 | 579 | 0 |  | NE | 0 | 579 | 0 |
| Diagnosis | 1 | 579 | 0.17 |  | Epinephrine | 0 | 579 | 0 |
| Hepatitis | 0 | 579 | 0 |  | Dopamine | 0 | 579 | 0 |
| ChildPH | 0 | 579 | 0 |  | Dobutamine | 0 | 579 | 0 |
| +UD | 0 | 579 | 0 |  | Reverse | 0 | 579 | 0 |
| DM | 0 | 579 | 0 |  | Extubation | 0 | 579 | 0 |
| HTN | 0 | 579 | 0 |  | hospstayd | 0 | 579 | 0 |
| DLP | 0 | 579 | 0 |  | Intubation~d | 0 | 579 | 0 |
| UDCardio | 0 | 579 | 0 |  | Postophosp~d | 0 | 579 | 0 |
| preHb | 0 | 579 | 0 |  | Reop | 0 | 579 | 0 |
| preHct | 0 | 579 | 0 |  | Deathinadm | 0 | 579 | 0 |
| preWBC | 0 | 579 | 0 |  | momortality | 0 | 579 | 0 |
| prePlt | 0 | 579 | 0 |  | Stroke | 0 | 579 | 0 |
| preBUN | 0 | 579 | 0 |  | delirium | 0 | 579 | 0 |
| preCr0 | 0 | 579 | 0 |  | Atelect | 0 | 579 | 0 |
| AKI | 0 | 579 | 0 |  | Pneumonia | 0 | 579 | 0 |
| AKIstage | 0 | 579 | 0 |  | Pleuraleff | 0 | 579 | 0 |
| Alb | 0 | 579 | 0 |  | PE | 0 | 579 | 0 |
| ALP | 0 | 579 | 0 |  | Hypotension | 0 | 579 | 0 |
| AST | 0 | 579 | 0 |  | Ileus | 0 | 579 | 0 |
| ALT | 0 | 579 | 0 |  | UTI | 0 | 579 | 0 |
| DB | 0 | 579 | 0 |  | SSI | 0 | 579 | 0 |
| TB | 0 | 579 | 0 |  | DVT | 0 | 579 | 0 |
| Anestimemin | 0 | 579 | 0 |  | ARDS | 0 | 579 | 0 |
| Optimemin | 0 | 579 | 0 |  | Liverfailure | 0 | 579 | 0 |
| crystalloid | 0 | 579 | 0 |  | CardiacArrest | 0 | 579 | 0 |
| Colloid | 0 | 579 | 0 |  | stageCKD | 0 | 579 | 0 |
| Stratch | 0 | 579 | 0 |  | CKD | 0 | 579 | 0 |
| Gelatin | 0 | 579 | 0 |  | HD | 0 | 579 | 0 |
| Albumin | 0 | 579 | 0 |  | POsepsis | 0 | 579 | 0 |
| PRCtrans | 0 | 579 | 0 |  | Pobleed | 0 | 579 | 0 |
| FFPtrans | 0 | 579 | 0 |  | posbalance | 0 | 579 | 0 |
| plttrans | 0 | 579 | 0 |  | eGFR0 | 0 | 579 | 0 |
| EBL | 0 | 579 | 0 |  | eGFR1 | 0 | 579 | 0 |
| UOPml | 0 | 579 | 0 |  |  |  |  |  |

**Comment 3.23: Clarify the surgical approach (open, laparoscopic, laparoscopic-assisted, or robotic) and discuss potential confounding effects on AKI incidence.**

Response: Thank you for pointing this out.

The surgical approach could indeed influence the incidence of acute kidney injury (AKI), as pneumoperitoneum and increased intrabdominal pressure in minimally invasive procedures may affect renal perfusion. For this reason, we included only open liver resections in our study to maintain consistency in surgical technique and avoid any potential confounding effects on AKI outcomes. Accordingly, we have modified the title of our paper to:

“The Impact of Perioperative Positive Fluid Balance on Postoperative Acute Kidney Injury in Patients Undergoing Open Hepatectomy: A Retrospective Single-Center Cohort Study.”

**Comment 3.24: Discuss the relationship between low BMI (<18 kg/m²) and AKI development, referencing relevant studies (e.g., DOI: 10.1097/ALN.0000000000005215). In the database, 91 patients had an extremely low body mass index of less than 18 kg/m2 and 35 patients had a BMI < 17 kg/m2, which typically indicates severe malnutrition and is associated with significantly increased perioperative risks, including impaired wound healing, infections, and AKI. Numerous studies report an extremely low BMI is indeed associated with a higher risk of developing AKI after surgery (see https://doi.org/10.1097/ALN.0000000000005215 and DOI: 10.1053/j.jrn.2023.01.005. Could the author comment on whether a low BMI is independently associated with AKI?**

Response: Thank you for pointing this out.

| Variables | Univariable analysis | | | Multivariable analysis | | |
| --- | --- | --- | --- | --- | --- | --- |
|  | OR | p | 95% CI | OR | p | 95% CI |
| BMI >25 kg/m^2^ | 0.66 | 0.235 | 0.34, 1.31 | 0.68 | 0.261 | 0.34, 1.34 |
| BMI <18 kg/m^2^ | 0.71 | 0.488 | 0.27, 1.85 | 0.65 | 0.375 | 0.24, 1.70 |
| Albumin < 2.8 g/dl | 1.39 | 0.510 | 0.52. 3.74 | 1.00 | 1.000 | 0.35, 2.84 |

In the current analysis, low BMI (<18 kg/m²) demonstrated a trend toward increased odds of acute kidney injury (AKI) development, though the association was not statistically significant in either the univariable (OR 0.71, 95% CI 0.27–1.85, p = 0.488) or multivariable analysis (OR 0.65, 95% CI 0.24–1.70, p = 0.375). These findings suggest that while low BMI may not have emerged as an independent predictor of AKI in this dataset, it remains an important variable to consider, especially given its association with malnutrition and perioperative risks.

Low BMI, particularly values indicative of severe malnutrition (<17 kg/m²), has been reported in the literature as a risk factor for adverse outcomes, including impaired wound healing, infections, and organ dysfunction such as AKI. For example, a study by Douville et al. (DOI: 10.1097/ALN.0000000000005215) demonstrated that underweight patients were more prone to AKI following surgery, likely due to reduced physiological reserves, impaired immune responses, and altered drug metabolism. Similarly, research by Lan J et al. (DOI: 10.1053/j.jrn.2023.01.005) highlighted the vulnerability of malnourished patients to renal injury due to reduced renal perfusion and increased susceptibility to ischemia-reperfusion injury.

The lack of statistical significance in this study may be attributed to the relatively small sample size of patients with extremely low BMI (<18 kg/m²) and even fewer (<17 kg/m²). Future studies with larger cohorts and adjusted for other confounding variables may better delineate the independent impact of low BMI on AKI risk. Additionally, evaluating nutritional status through alternative or complementary indices, such as preoperative nutritional screening tools, could provide further insights into the role of malnutrition in perioperative AKI.

**Comment 3.25: A detailed descriptions of the type/s of colloids used needs to be reported. Certain colloids are strongly associated with the development of postop operative AKI. Some large studies with > 44,000 surgical patients show a dose-dependent renal toxicity with starch with 21% greater risk of developing AKI vs. crystalloid. See DOI: 10.1097/ALN.0000000000000375**

Response: Thank you for pointing this out.

|  | Propensity score matching | | | |
| --- | --- | --- | --- | --- |
| Variables | < 1 L Group  (n=193) | 1-2 L Group  (n=193) | > 2 L Group  (n=193) | p |
| Colloid usage | 105 (54.4) | 133 (68.9) | 179 (92.8) | <0.001 |
| Starch usage | 89 (46.1) | 117 (60.6) | 157 (81.4) | <0.001 |
| Gelatin usage | 16 (8.3) | 23 (11.9) | 59 (30.6) | <0.001 |

| Variables | Univariable analysis | | | Multivariable analysis | | |
| --- | --- | --- | --- | --- | --- | --- |
|  | OR | p | 95% CI | OR | p | 95% CI |
| Colloid usage | 2.13 | 0.028 | 1.08, 4.18 | 1.47 | 0.293 | 0.71, 3.07 |
| Starch usage | 1.45 | 0.182 | 0.84, 2.53 | 1.04 | 0.893 | 0.57, 1.91 |
| Gelatin usage | 1.64 | 0.109 | 0.89, 3.03 | 1.09 | 0.814 | 0.54, 2.17 |

In our study, the colloids used were primarily starch-based solutions and gelatin-based solutions. The univariable analysis indicates that colloid usage is significantly associated with increased odds of developing AKI (OR 2.13, 95% CI 1.08–4.18, p = 0.028). However, this association loses statistical significance in the multivariable analysis (OR 1.47, 95% CI 0.71–3.07, p = 0.293), suggesting that other factors may confound or mediate this relationship. For starch and gelatin usage, neither univariable nor multivariable analyses show significant associations with AKI, with p-values well above the threshold for significance.

The findings from large-scale studies, such as the one referenced (DOI: 10.1097/ALN.0000000000000375), demonstrate a dose-dependent renal toxicity with starch and a significantly higher risk of AKI compared to crystalloids. The absence of a significant association in our study may be attributed to differences in starch formulations, lower dosages used, or patient selection.

**Comment 3.26: There are numerous typographical errors and errors with syntax that need to be corrected. A professional editing service would substantially enhance this manuscript and improve its competitiveness for publication in PLOS ONE. Consider services such as https://www.medicaljournaleditors.com. This is an excellent service that provides comprehensive editing services. Alternative services include Editage (https://www.editage.com)**

Response: Thank you for pointing this out.

In addition to the comments, all spelling and grammatical errors have been corrected again by the staff of the Chiang Mai university English language team (CELT) for language editing assistance.

**Comment 3.27: Revise or delete the first paragraph of the discussion, as it does not align with the study findings.**

Response: Thank you for pointing this out. We updated the manuscript according to your recommendations.

**Comment 3.28: Structure the discussion into the following sections for coherence:**

**o Key findings (1 paragraph)**

**o Relationship to existing literature (2–3 paragraphs)**

**o Clinical implications (2 paragraphs)**

**o Strengths and limitations (2 paragraphs)**

**o Conclusions (1 paragraph)**

Response: Thank you for pointing this out. We have revised the entire discussion based on your advice.

**Comment 3.29: Discuss potential mechanisms linking excessive fluid administration (>2L) with increased AKI risk.**

Response:

Excessive fluid administration, particularly volumes exceeding 2 liters, has been associated with an increased risk of acute kidney injury (AKI) through several pathophysiological mechanisms. One key mechanism is hypervolemia, which elevates intravascular hydrostatic pressure, triggering the release of atrial natriuretic peptides. While atrial natriuretic peptides play a role in regulating fluid balance, their overactivation can have detrimental effects, including damage to the endothelial glycocalyx.

The glycocalyx is a protective, gel-like layer lining the endothelium, crucial for maintaining vascular integrity and permeability. Damage to this layer compromises its barrier function, leading to increased vascular permeability and fluid leakage into surrounding tissues. In the kidneys, the glycocalyx on endothelial cells within glomerular capillaries is vital for key processes such as filtration and reabsorption. Disruption of the renal glycocalyx can impair these functions, resulting in glomerular injury, reduced filtration efficiency, and tubular dysfunction. This cascade of events may ultimately contribute to AKI development, highlighting the critical balance required in fluid management to avoid both hypoperfusion and hypervolemia-induced complications.

**Comment 3.30: Revise the conclusion for clarity. Consider the following “This retrospective study of 691 hepatectomy patients suggests that maintaining a fluid balance of 1–2 liters at surgery’s end minimizes AKI risk. Fluid excess or liberal fluid strategies were associated with renal impairment and AKI. Additional risk factors for AKI included CTP class B/C cirrhosis, prolonged operative duration, and significant blood loss.”**

Response: Thank you for pointing this out. We updated the manuscript according to your recommendations.

We look forward to hearing from you in due time regarding our submission and to responding to any further questions and comments you may have.

Sincerely, yours

Natsuda Phothikun
Department of Anesthesiology, Faculty of Medicine,
Chiang Mai University, Chiang Mai, Thailand
110 Intravaroros Road, Sriphum, Mueng,
Chiang Mai 50200, Thailand

Email: natsuda.dkptk@gmail.com
Tel: +66849090104
